# New migration and distribution patterns of Atlantic walruses (*Odobenus rosmarus rosmarus*) around Nunavik (Québec, Canada) identified using Inuit Knowledge

**DOI:** 10.1007/s00300-021-02920-6

**Published:** 2021-08-06

**Authors:** Laura M. Martinez-Levasseur, Chris M. Furgal, Mike O. Hammill, Dominique A. Henri, Gary Burness

**Affiliations:** 1 Department of Biology, Trent University, Peterborough, ON K9L 0G2 Canada; 2 Indigenous Environmental Studies & Sciences Program, Trent University, Peterborough, ON K9L 0G2 Canada; 3 Wildlife Research Division, Environment and Climate Change Canada, Montréal, QC H2Y 2E7 Canada; 4 Maurice Lamontagne Institute, Fisheries and Oceans Canada, Mont-Joli, QC G5H 3Z4 Canada

**Keywords:** Traditional Ecological Knowledge, Local Ecological Knowledge, Inuit communities, Atlantic walruses, *Odobenus rosmarus rosmarus*, Canadian Arctic, Nunavik

## Abstract

Environmental changes are affecting the Arctic at an unprecedented rate, but limited scientific knowledge exists on their impacts on species such as walruses (*Odobenus rosmarus*). Inuit Traditional and Local Ecological Knowledge (Inuit TEK/LEK) held by Inuit walrus harvesters could shed light on walrus ecology and related environmental changes. Our main objective was to study spatial and temporal changes in Atlantic walrus (*Odobenus rosmarus rosmarus*) distribution in Nunavik (northern Québec, Canada) using Inuit TEK/LEK. To do so, we documented the knowledge and observations of 33 local hunters and Elders as part of a larger project on Atlantic walruses in Nunavik. We first gathered information on changes in Inuit land use patterns and harvesting practices through time and space, which was a crucial step to avoid potential biases in interpreting local observations on walrus distribution. We found that walrus hunters are now covering smaller hunting areas over shorter time periods, reducing in space and time their observations of Atlantic walruses around Nunavik. While clearly taking these limitations into account, we learned from interviews that some areas abandoned by Atlantic walruses in the past were now being re-occupied. Importantly, Atlantic walruses, which migrate following the melting ice, are now traveling along the eastern coast of Nunavik one month earlier, suggesting that Atlantic walrus migration has changed due to variations in sea-ice coverage around Nunavik. Our study not only highlighted important changes in Atlantic walrus distribution and migration in Nunavik, but also sheds light on the importance of documenting temporal and spatial changes in Inuit land use patterns and harvesting practices to understand the ecology of Arctic species using Inuit Knowledge.

**Supplementary Information:**

The online version contains supplementary material available at 10.1007/s00300-021-02920-6.

## Introduction

Walruses, *Odobenus rosmarus*, have a discontinuous circumpolar Arctic and sub-Arctic distribution, and are represented by two subspecies: the Pacific walrus, *Odobenus rosmarus divergens*, which occurs in the Arctic and sub-Arctic waters of the Chukchi, Bering and Laptev seas (USA and Russia) (Fay 1982; Lindqvist et al. 2009) and its abundance has been estimated above 200,000 (Lowry 2016), and the Atlantic walrus, *Odobenus rosmarus rosmarus*, which inhabits coastal areas in the north-eastern Canada, Greenland (Denmark), Svalbard (Norway), and the Barents and Kara seas (western part of Arctic Russia) (Born et al. 2001), and its abundance has been estimated above 25,000 (Kovacs 2016; Lowry 2016). Although the IUCN (International Union for Conservation of Nature) and the IUCN SSP (Species Survival Commission) Pinniped Specialist Group has recommended Atlantic walruses to be listed as “Near Threatened” because of uncertainties surrounding future decline in their habitat quality, and because of the lack of data on general walrus abundances and trends (Lowry 2016), Inuit consider Atlantic walruses not at risk (DFO 2013).

In the Inuit region of Nunavik (northern Québec, Canada), Atlantic walruses are considered to be part of two different stocks: the stock of Southern and Eastern Hudson Bay (hereafter SE Hudson Bay stock) and the stock of Northern Hudson Bay–Hudson Strait–South-eastern Baffin Island–Northern Labrador (hereafter Hudson Strait stock) (see map in Stewart 2008). So far most research on Canadian Atlantic walruses has been conducted in Canada’s High Arctic (Stewart 2008), excepted from one aerial survey conducted around Nunavik in September 2014, which revealed an abundance estimate of 7,100 walruses within the Hudson Strait stock (Hammill et al. 2016).

**Fig. 1:**
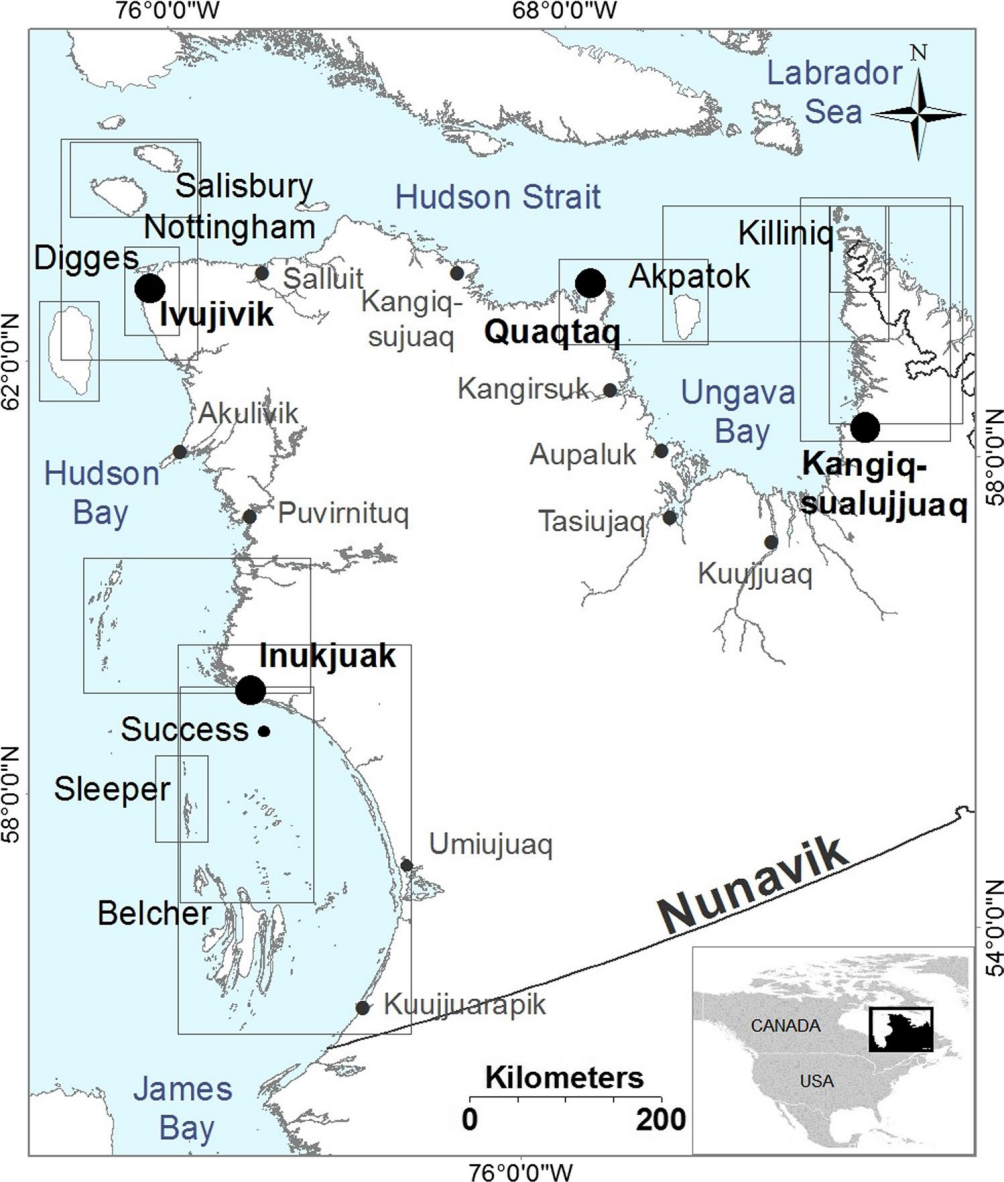
Map of Nunavik (Northern Québec, Canada), showing the four communities involved in the project (Inukjuak, Ivujivik, Quaqtaq, and Kangiqsualujjuaq). The quadrats show the limits of the base maps that were used during interviews and validation workshops to gather and then confirm spatial data of Atlantic walrus (*Odobenus rosmarus rosmarus*) distribution

Due to rapid environmental changes, there is a pressing need to increase our understanding of the distribution and ecology of Atlantic walruses, including those living around Nunavik. Negative impacts induced by environmental changes have already been reported for walruses and other marine mammals living across the Arctic. Around Alaska, Pacific walruses’ behavior, distribution, and mortality have been affected by a decrease in their sea-ice habitats (MacCracken 2012). In the same region, unusual sea-ice patterns were found to alter the migration course of belugas (*Delphinapterus leucas*), as well as their summer habitat use (O’Corry-Crowe et al. 2016). On the western side of Nunavik within Hudson Bay (Fig. 1 in methods), longer ice-free periods resulting from unusual melting sea-ice have coincided with high level of stress, low body condition, and low pregnancy rates in ringed seals (*Pusa hispida*) (Ferguson et al. 2017).

From a science perspective, past changes in wildlife distribution can be observed by analyzing long-term scientific datasets. However, such datasets rarely exist for species living in remote areas such as the Arctic. In this context, changes in arctic wildlife distribution and migration can be studied using rigorous Traditional and Local Ecological Knowledge (TEK/LEK) methods (Huntington 2000; Seidman 2006; Creswell 2009; Furgal and Laing 2012; Martinez-Levasseur et al. 2017). Indeed, TEK/LEK gathered through interviews with harvesters living in close relationship with wildlife is a key source of information for understanding the ecology and distribution of arctic wildlife (Huntington 2011; Service et al. 2014; Pardo-de-Santayana and Macía 2015; Breton-Honeyman et al. 2016), including walruses (Krupnik and Ray 2007; Metcalf and Robards 2008; Kowalchuk and Kuhn 2012; Martinez-Levasseur et al. 2016, 2020).

For thousands of years, walruses have been hunted by northern Indigenous communities, which have relied on walruses and other marine mammal species for survival in the Arctic (Krupnik and Ray 2007). Walruses continue to play an important role among northern indigenous communities, although they no longer form a significant portion of annual indigenous diet (Council of Canadian Academies 2014). Moreover, since the nineteenth century, Inuit land use patterns and harvesting practices have changed. In Nunavik, most of these changes have occurred since the Hudson’s Bay Company established a trading post in 1830 at the present location of Kuujjuaq (Fig. 1) (Dorais 1997). At the beginning of the twentieth century, metal had mostly replaced the traditional materials (stone, bone, and ivory) used among Inuit for making tools and weapons (Krupnik and Ray 2007). By 1915, the use of hunting firearms was common in Nunavik. The multiplication of trading posts and the establishment of stores which provided, among other goods, firearms, ammunitions, and gasoline, encouraged Inuit into permanent settlement. For example, Quaqtaq (Fig. 1) evolved from a winter camp in the 1940s–1950s to a permanent village in the 1960s, particularly when social and educational services were established (Dorais 1997). Between the 1940s and the 1960s, Inuit continued to use dog teams, kayaks, and peterhead boats to hunt, fish, and trap. Dog teams used to be an integral part of Inuit culture, daily life, and survival (Qikiqtani Inuit Association 2014). But around the end of the 1960s, the use of outboard motor canoes and snowmobiles (Qikiqtani Inuit Association 2014) changed the way Inuit traveled, which accelerated the decline of spring camps because Inuit could reach their hunting grounds and come back within a day (Dorais 1997). By 1990, most Inuit from Nunavik were familiar with these technologies (Dorais 1997).

Our study objectives were to (1) understand how changes in Inuit land use patterns and harvesting practices have modified the locations and sizes of Atlantic walrus hunting areas, the time spent by hunters looking for Atlantic walruses in those areas, and the number of Atlantic walruses harvested; and to (2) understand if the distribution and migration patterns of Atlantic walruses have changed around Nunavik. We particularly wanted to explore whether the significant variations in sea-ice patterns occurring since few decades in the eastern part of Nunavik (Quaqtaq area) have induced spatial and temporal changes in Atlantic walrus distribution and migration in this region. To do so, we analyzed the qualitative and spatial data obtained through 33 interviews realized with Inuit walrus hunters and Elders as part of a larger project on Atlantic walruses in Nunavik (Martinez-Levasseur et al. 2016, 2017).

## Methods

This study is part of a larger project entitled “Walruses and population health in Nunavik, drawing upon both scientific methods and local ecological knowledge” (Martinez-Levasseur et al. 2016, 2017, 2020), which was approved by the four participating Inuit communities and their local Hunting Fishing and Trapping Associations, Northern Villages and Landholding Corporations (March–September 2013), the Nunavik Marine Region Wildlife Board (December 2012), the Trent University Research Ethics Board (December 2012), and the Trent Aboriginal Education Council Ethics Review Committee (February 2013). The corresponding methods used, including participant selection, questionnaire, interviews, spatial data collection, and internal validation workshops, are discussed in detail in Martinez-Levasseur et al. 2017. Standards for social research methods used to document local and traditional knowledge were followed (Huntington 2000; Seidman 2006; Creswell 2009; Furgal and Laing 2012).

### Defining Inuit Traditional and Local Ecological Knowledge (Inuit TEK/LEK)

Ecological knowledge held by Indigenous peoples is often referred as traditional ecological knowledge, or TEK, which can be conceptualized as a practice–knowledge–belief system focused on ecological relationships (Berkes et al. 2000; Huntington 2011; Berkes 2012). Some studies have also begun to use the term local ecological knowledge, or LEK, to describe the knowledge corresponding to observational information acquired over participant lifetimes (Davis and Wagner 2003; Gilchrist et al. 2005; Furgal and Laing 2012; Martinez-Levasseur et al. 2016, 2017). In our study, we employed the expression Traditional and Local Ecological Knowledge (TEK/LEK) as we reported observations made by participants over their lifetime, as well as knowledge transmitted over generations. Given that participants included 32 Inuit hunters and Elders, as well as one non-Inuk hunter, we deemed appropriate to refer to Inuit TEK/LEK to refer to the ethnicity of the vast majority of participants.

### Area of study and participant selection

The communities of Inukjuak, Ivujivik, Quaqtaq, and Kangiqsualujjuaq in Nunavik situated in Northern Québec, Canada (Fig. 1) were selected to participate in this study due to their active participation in walrus hunting (Larrat et al. 2012), wide geographic distribution representing the entire range of walruses in Nunavik (Stewart 2008), and their interest in Inuit knowledge–science research as evidenced by active participation in previous projects (Breton-Honeyman et al. 2016). Ten to 15 walrus hunters and Elders were identified in each community and invited to participate in the study during our first visit to communities (March and June 2013). These walrus hunters and Elders were recognized for their knowledge and participation in walrus hunts by the local Hunters, Fishers, and Trappers Associations and other community members. Note that although the communities selected are based in Nunavik, participants provided information on observations of walruses in both Nunavik and Nunavut (as many islands around Nunavik are part of the Nunavut territory).

### Interviews and spatial data collection

Semi-directive interviews were conducted between June and September 2013 (for details see Martinez-Levasseur et al 2017). During interviews, we gathered spatial data on harvesting areas and walrus distribution using 10 printed base maps (quadrats in Fig. 1) in both English and Inuktitut of the areas surrounding participating communities. These maps were created using the geographic information system software ArcMap 10.4.1. and digital vector datasets of coastlines and islands from the RNCan-National Topographic Database. Map scales varied between 1:100,000 and 1:450,000, depending on the extent of walrus hunting areas provided by the local Hunters, Fishers, and Trappers Associations during our first visit. A regional map (scale: 1:2,000,000) was also created. The mapping process that took place during the interviews followed guidelines previously described (Tobias 2009; Martinez-Levasseur et al. 2017). Participants drew points (e.g., walrus kill sites), lines (e.g., walrus migration routes, boat trajectories), or polygons (e.g., areas where walruses had been observed) onto transparent plastic overlays covering base maps. Each point, line or polygon drawn by participants was attributed an alphanumeric code that facilitated the reconnection of audio-recorded information with maps (i.e., data such as the date and source of observation) (see details in Martinez-Levasseur et al. 2017). Before the interview, participant consent form and letter of information (summary of the project) were provided to each participant in English or Inuktitut (at the participant’s preference).

### Qualitative data analysis and internal validation

The audio-recorded interviews were transcribed, entered into the qualitative analytical software program NVivo10 (Version 10, 2012), and analyzed using thematic content analyses (Creswell 2009). The 58 maps created during the interviews were scanned, digitized, and analyzed using ArcMap 10.4.1. The maps created (by season and/or time period) (Gadamus and Raymond-Yakoubian 2015) combined the personal knowledge of all participants, as well as, in some cases, knowledge transmitted to participants by their ancestors. To verify and validate preliminary results with participants, internal validation workshops were organized during a third visit to communities in July 2014 (Martinez-Levasseur et al. 2017). Only those geographic areas and data (e.g., number of walruses) that could be validated during workshops were included in subsequent analyses. The few spatial observations removed, mostly isolated data, corresponded to errors, as pointed out by participants present at the workshops. These workshops not only allowed the validation or correction of our interpretation of the data, but also kept participants informed about the progress of the study. Finally, following further analyses, results were presented in each participating community in March 2015. This final trip was used to share final results and distribute corresponding reports with participants, local organizations, and other community residents.

### Participants’ temporal and spatial limits of observations

A common bias that can occur when looking at Inuit TEK/LEK maps is for marine animals to appear concentrated along the coast, when in fact it is the observations of Inuit hunters that are concentrated along the coast (Martinez-Levasseur et al. 2017). Similarly, animals may appear to exhibit distributional changes over time, when in fact it is the Inuit harvesting areas that have changed over time. To avoid these biases, we defined for each time period and season the geographic limits of participants’ common areas of observations (Martinez-Levasseur et al. 2017). Participants who were present during the group validation workshops were asked to draw the geographic areas they were familiar with, and for which they had direct observational knowledge and experience for specific time periods and seasons. Geographic limits of participants’ common areas of observations were (1) community specific, (2) season specific, and (3) time-period specific (details in Martinez-Levasseur et al. 2017).

### Dataset

In total, 33 local walrus hunters and Elders participated in the study, corresponding to 55% of the potential participants identified by the local Hunters Fishers and Trappers Associations during the first visit. In each community, we interviewed between 7 and 10 participants (10, 8, 8, and 7 participants in Quaqtaq, Ivujivik, Kangiqsualujjuaq, and Inukjuak, respectively). Participants ranged in age from 35 to 85 years old, thus providing observations extending back to the 1940s. Among the 33 interviewees, four women and one non-Inuk hunter recognized as a walrus expert by the local Hunters Fishers and Trappers Association were included. Of the participants interviewed, 69% subsequently participated in the validation workshops. Seasons were defined and confirmed by participants as fall (September–mid-December), winter (end of December–April), spring (May–mid-June), and summer (end of June–August).

In the analyses and presentations of results, we demarcated three time periods in the history of walrus observations and hunting in Nunavik: 1940s–1990s, 2000s–2010s (both within participants’ lifetimes), and the knowledge held by participants’ ancestors that we defined as prior to the 1940s. After rigorously defining areas and time periods for which walrus hunters have accumulated knowledge on walrus distribution and movements (objective 1), we documented participant-identified changes in walrus distribution and movements around Nunavik (objective 2). Indeed, understanding the spatial and temporal range of participant observations is crucial for data interpretation.

Finally, when we used fractions to represent results (e.g., 12 out of 17 participants reported that walruses migrate earlier), it means—using the example provided—that 17 participants were asked the question (“Since you first hunted walrus, have you seen changes in the dates when walruses are arriving around your community?,” and that, 12 participants answered yes. The other participants (5 out of 12) answered no that they haven’t notice change or answered that they did not know.

## Results

It is important to note that the knowledge presented in this study does not represent all Nunavik Inuit Knowledge on walrus harvesting and distribution but only the knowledge of 33 hunters and Elders, as gathered in the context of this study.

### Objective 1: Changes in Inuit land use patterns and harvesting practices

#### Faster boats

David Okpik, an Elder from Quaqtaq who was born in the 1930s, explained that in the past Inuit were making observations from specific elevated points, corresponding to hills along the coast, looking for Atlantic walruses and checking currents before leaving by boat. *“Now it’s diminished.* […] *It’s much faster by boat* [outboard motor canoe]*, so fast that you just go* [without checking first]*”*. Nowadays, two types of motor boats are used for walrus hunting: peterhead boats (used to reach far-off walrus hunting areas) and outboard motor canoes.

#### Fewer Atlantic walruses harvested

All participants reported that fewer walruses are harvested today because Inuit have fewer sled dogs (replaced by snowmobiles in the 1960s) to feed. Ivujivik participants explained that nowadays they select more carefully which walrus to hunt as their catch are now mainly intended for human consumption, whereas before it used to be mainly for dogs. Inukjuak participants also added that today less walrus hunting occurs due to the high costs of hunting.

#### Smaller hunting areas and shorter hunting periods

Changes in the locations of walrus hunting areas and the duration of the hunts were reported for the 1940s–2010s period. Participants reported that, since 2000s, walrus hunting areas have become smaller in size and the time spent to hunt walruses is now shorter. Consequently, the geographic areas and the time scale for which walrus hunters can provide detailed information have been reduced. This is particularly true for hunters from Quaqtaq, Ivujivik, and Inukjuak, who are specialist walrus hunters (i.e., planning hunting trips specifically to harvest walruses), whereas Kangiqsualujjuaq hunters are opportunistic (i.e., while doing another activity, they can harvest a walrus).

Note that while participants from Quaqtaq have been hunting and observing Atlantic walruses from the Hudson Strait stock during their spring migration (end of June to mid-July), participants from Ivujivik have been harvesting the same stock of walruses near their resting grounds around Nottingham and Salisbury Islands in the fall (September–October) (see details on walrus migration in Online Resource ESM-2). During the same period, Inukjuak hunters have been hunting Atlantic walruses from the SE Hudson Bay stock. Combining information from these four communities allowed us to cover the entire range of Atlantic walruses in Nunavik (Stewart 2008). Specific results from each participating community are presented below.

**Fig. 2:**
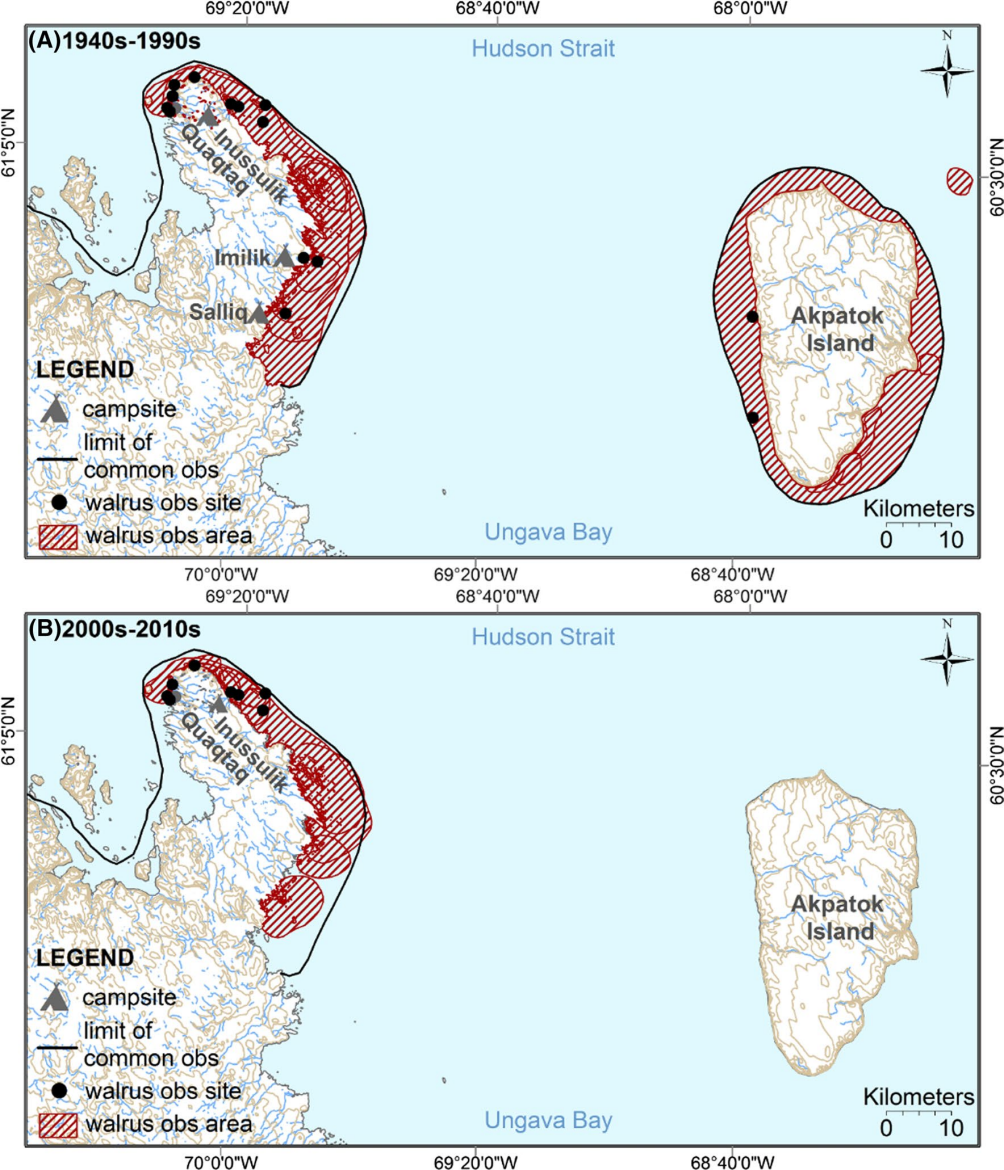
Distribution of Atlantic walruses (*Odobenus rosmarus rosmarus*) during summer based on Inuit knowledge and observations for the 1940s–1990s period (observations made mostly in August) (**A**) and for 2000s–2010s (observations made mostly during the first 2 weeks of July) (**B**). Each hatched area and point were drawn by one participant and corresponds to areas or sites where walruses had been observed by this participant. The black lines correspond to the limits of the common areas of observations of all participants. These maps combined the digitalized data collected from nine Quaqtaq participants in 2013–2014

*Quaqtaq *Until the 1990s, Quaqtaq walrus hunters waited until the ice melted in August to travel to Akpatok Island (a 4-hour trip by peterhead boat) to hunt walruses (Fig. 2a). Paul Jararuse from Kangiqsualujjuaq explained that “*in the past* [earlier than the 1930s], *any community from Ungava Bay used to hunt walruses on Akpatok Island by sailing boat*.” For the 1940s–1990s period, Quaqtaq hunters also explained that they used to stay for several weeks in their summer camps around *Imilik* and *Salliq* islands near the coast of Ungava Bay (Fig. 2a), where they also hunted walruses. Although nowadays hunters continue to travel as far as those islands, which are about 2 h away by motor canoe from Quaqtaq, hunters generally prefer to hunt walruses during day trips close to the community, which increases their chance of getting help from other hunters for butchering their catch. Since the 2000s, Quaqtaq walrus hunters have stopped accumulating knowledge on walrus distribution and movement around Akpatok Island. Nowadays, most walrus observations and kills occur along the north-west coast of Ungava Bay (Fig. 2b) during walrus spring migration (end of June to mid-July) because the ice now melts earlier, explained participants.

**Fig. 3:**
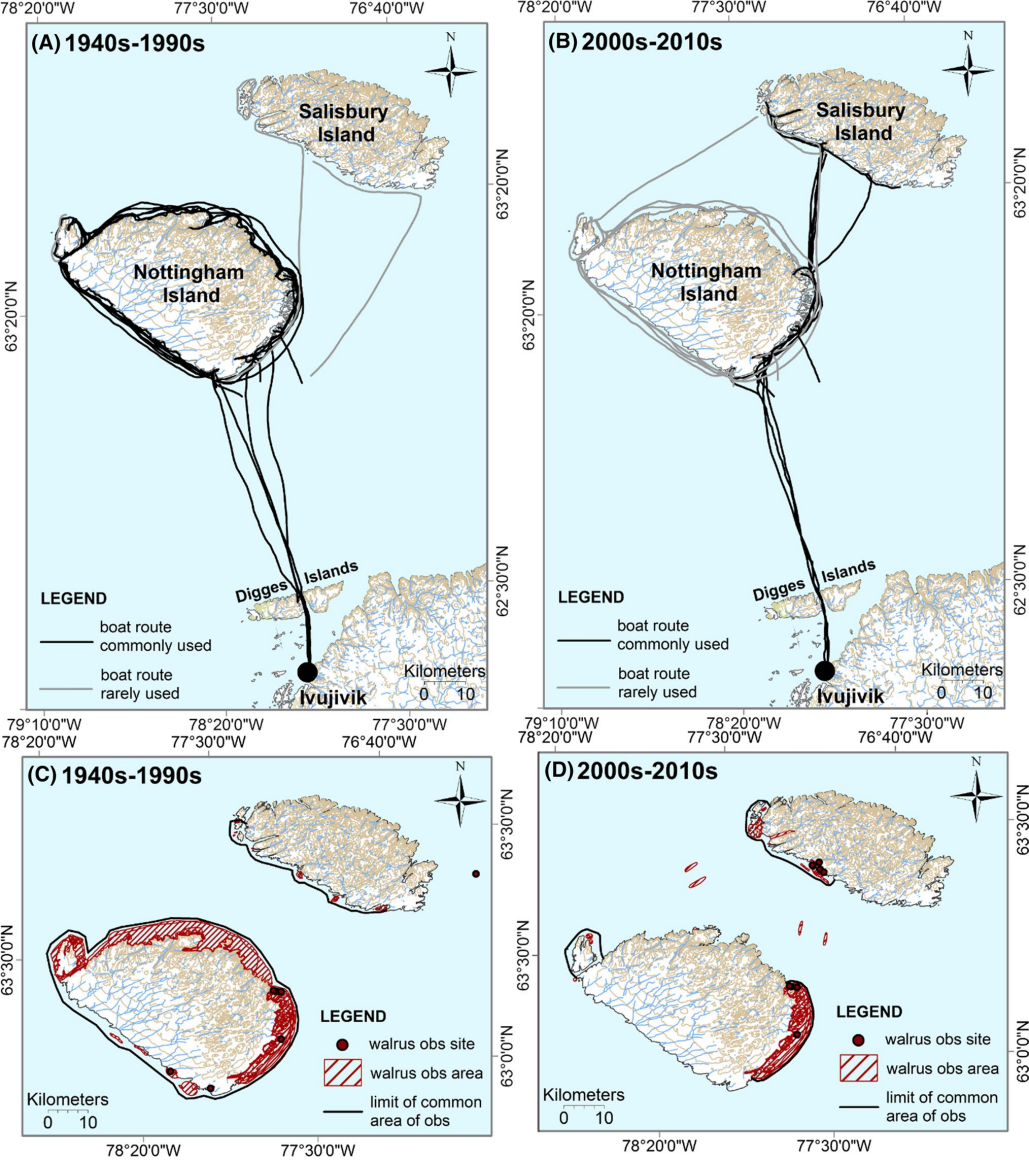
Routes taken by the community boat of Ivujivik to go Atlantic walrus (*Odobenus rosmarus rosmarus*) hunting in the fall (mainly in September) in the 1940s–1990s (**A**, **C**) and in the 2000s–2010s (**B**, **D**). Each line (**A**, **B**) was drawn by one participant and represents one of the paths taken by the community boat to go walrus hunting. A single participant could draw different lines if the yearly walrus expeditions he followed took different routes. Each hatched area and point (**C**, **D**) was drawn by one participant and corresponds to areas or sites where walruses were observed or harvested by this participant. The black lines (**C**, **D**) correspond to the limits of the common areas of observations of all participants. This figure combines the digitalized data collected from eight Ivujivik participants in 2013–2014

*Ivujivik* Prior to the 1990s, Ivujivik walrus hunters used to boat around Nottingham Island (*Tujjaat*) in the fall (generally in September) to look for Atlantic walruses (Fig. 3a). However, since the 2000s, if hunters did not see walruses on the south-east of Nottingham Island (which can be reached in one day by peterhead boat from Ivujivik), they would cross to Salisbury Island (*Akulliq*) (Fig. 3b). Indeed, their chances of finding walruses in the south-west of Salisbury were higher than around Nottingham Island, explained participants. Furthermore, communication devices such as satellite phones now allow hunters from different boats to communicate with each other, and adjust their routes to find walruses faster. By increasing their chance to find walruses, hunters save gas. As a whole, the length of walrus hunting trip has been reduced for Ivujivik harvesters to approximately 1 week instead of one month, as was the case prior to the 1990s. Since the 2000s, Ivujivik walrus hunters have stopped accumulating knowledge on walrus distribution along the entire coast of Nottingham Island. Nowadays, they mainly accumulate knowledge on walrus distribution on the south-east part of Nottingham Island and the south-west part of Salisbury Island over the course of 1 week in September mostly.

*Inukjuak* Ancestors of Inukjuak hunters used to hunt walruses on Success Islands (*Kikirtaaruk*) in summer and Belcher Islands (*Sanikiluaq*) in the fall (Fig. S1 in Online Resource ESM1). Atlantic walruses were also observed in the fall by participants’ ancestors on King George Islands (Fig. S1 in Online Resource ESM1). Those areas are not used anymore for walrus hunting, providing further evidence that hunting practices are constantly changing over time.

Between the 1940s and 1990s, Inukjuak hunters harvested walrus in the fall, mainly during the months of September and October, around Sleeper Islands (*Quumiutait*) (Fig. S1 in Online Resource ESM1), which took a day to reach by peterhead boat from Inukjuak. Since the mid-2000s, Inukjuak hunters have been harvesting Atlantic walruses around Nottingham and Salisbury Islands (*Tujjaat* and *Akulliq*) (Fig. 1), which take approximately one week to reach by peterhead boat. Such a change in hunting location at first appears surprising, especially given the increased costs of fuel and safety risk raised by participants. However, 5 out of 6 Inukjuak participants explained that such change in hunting location results from the high frequency of walruses harvested around the Sleeper Islands tested positive for the zoonotic parasite *Trichinella nativa*. Indeed, one participant explained that if walrus meat is infected by this parasite, it cannot be eaten, which is a waste of meat, time, and money. *“Hunters used to go walrus hunting in Sleeper Islands but walruses are now infected with disease and as a result they have to go to Nottingham Island. There is no Trichinella in the Nottingham walruses,”* explained Simeonie Ohaituk from Inukjuak. Since the 2000s, Inukjuak walrus hunters have stopped accumulating knowledge on walrus distribution and movements around the Sleeper Islands because of concerns about zoonotic diseases. On the contrary, they now accumulate knowledge on walrus distribution and movements around Nottingham and Salisbury Islands in September mostly.

### Objective 2: Understand if the distribution and migration patterns of Atlantic walruses have changed around Nunavik

#### Areas that have been abandoned by walruses

Before the 1940s, ancestors of Inukjuak participants hunted on Success Islands (*Kikirtaaruk*) in summer (Fig. S1 in Online Resource ESM1). Two participants from Inukjuak explained that there are close to 100 Atlantic walrus skulls left on these islands by their ancestors. They explained that, in the past, hunters used to leave the skulls of the walruses killed on site. They added that today walruses are no longer on Success Islands, an area that is part of their current common area of observations. When asking about the reason behind this change in distribution, one participant explained that species move from one place to another to look for food.

#### Areas where walruses have returned

The ancestors of Ivujivik participants used to harvest walruses on the western island of the Digges Islands Archipelago (*Saarqajaaq*) (Fig. S2 in Online Resource ESM1). Although walruses had not been observed on this island for decades, hunters began observing them basking there again since 2000 (Fig. S2 in ESM1). For example, in October 2013, hunters saw around 23 walruses basking on the north-western shores of the archipelago (Fig. S2 in Online Resource ESM1).

Ivujivik participants explained that the western island of the Island of Digges Islands Archipelago (*Saarqajaaq*) was abandoned by walruses because their ancestors had disturbed the walruses resting there by killing them directly on the land, instead of killing them while they were swimming. *“In the past our forefathers, our ancestors, were hunting walruses on the island of Saarqajaaq. Nowadays, it has been abandoned by the walruses.* […] *Those islands are termed Ulliviniq, which means it used to be a basking area, but it has been abandoned.* […] *There is an understood law, it is a kind of a rule among the hunters, to not disturb the walruses while they are on the Ulliq, basking on the island, do not disturb them, not to hunt them, not to kill them, so that the walruses will be able to return later to that same island, and not abandon it altogether as it has happened in the past,”* explained Quitsaq Tarriasuk from Ivujivik. This rule of not killing walrus on land was confirmed by the other seven Ivujivik hunters interviewed.

**Fig. 4:**
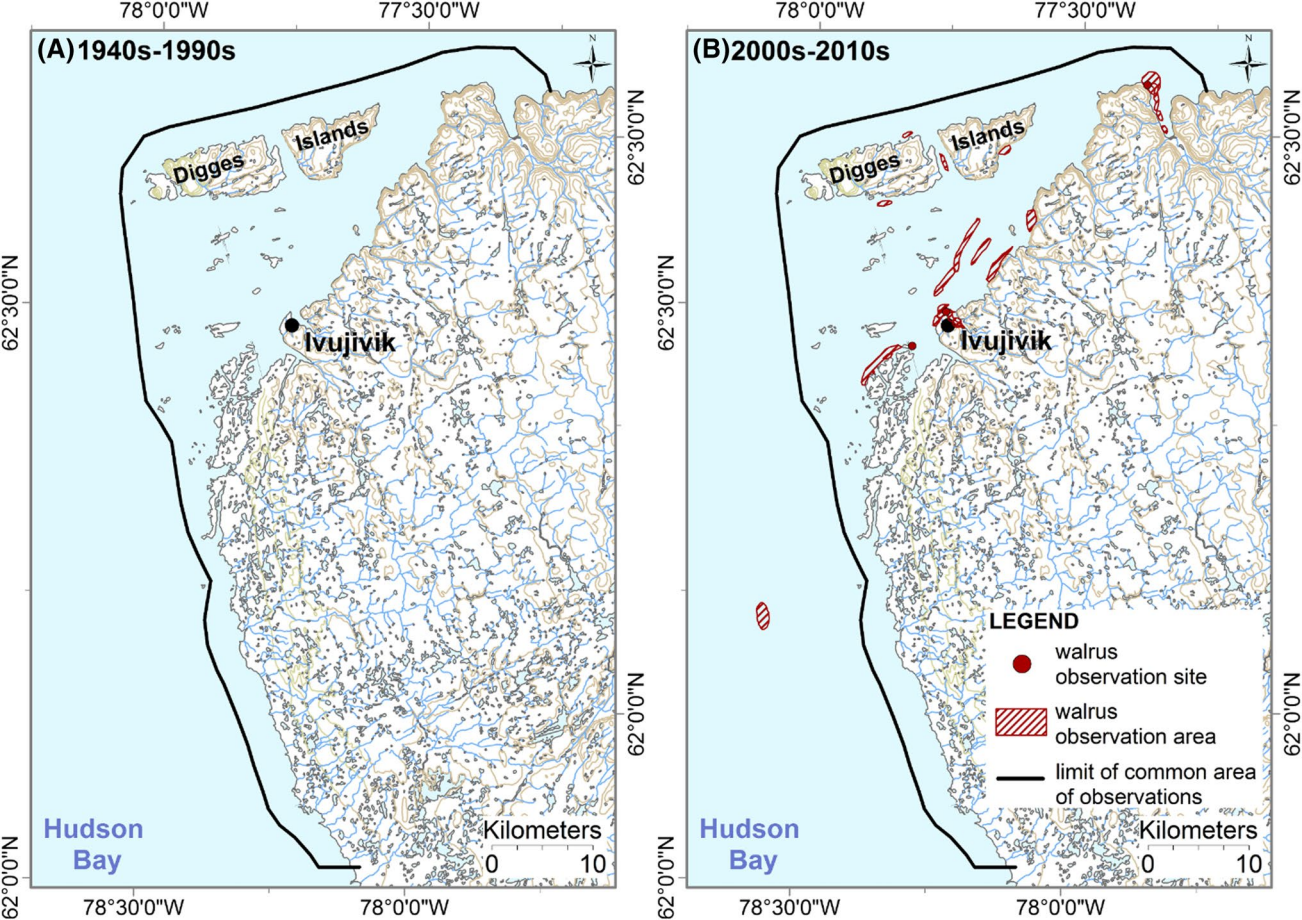
Distribution of Atlantic walruses (*Odobenus rosmarus rosmarus*) during summer based on Ivujivik Inuit knowledge and observations (**A**) for the 1940s–1990s period, and (**B**) the 2000s–2010s period. Each hatched area and point has been drawn by one participant and corresponds to areas or sites where walruses have been observed or harvested by this participant. The black lines correspond to the limits of the common areas of observation of all participants. Walrus observations made outside the participants’ common areas of observation on panel **B** correspond to one observation of three walruses traveling on floating ice for which the exact location was reported to be unsure. This figure combines the digitalized data collected from eight Ivujivik participants in 2013–2014

Finally, since the 2000s, Ivujivik participants have been observing more Atlantic walruses between Ivujivik and the Digges Islands during summer when compared with pre-1990 levels (Fig. 4).

### Atlantic walruses migrate around Nunavik one month earlier

In this study, only Inuit hunters and Elders from Quaqtaq and Kangiqsualujjuaq hunting in the area of Quaqtaq (Fig. 2) could provide information on potential changes in the timing of migration of Atlantic walruses. Indeed, these participants had been using the same marine mammal harvesting areas over their lifetime, and these areas corresponded to one of the spring northward migration routes taken by Atlantic walruses (see details on walrus migration in Online Resource ESM-2).

In total, 12 out of 17 participants from Quaqtaq and Kangiqsualujjuaq participants reported that Atlantic walruses are migrating earlier than in the past. Among Quaqtaq participants, 8 out of 9 explained that while today the walrus hunt occurs mostly during the first 2 weeks of July (along the north-west coast of Ungava Bay), the hunt used to occur in August before the 1990s (around Akpatok Island). Participants explained that Ungava Bay used to be frozen until August, and they could not travel by boat until the ice had cleared. “*We used to hunt in August because we used to have ice until August. Walruses migrate* [northward] *earlier now because there’s less ice*,” explained Johnny Oovaut from Quaqtaq. *“The walrus migration used to be in August. I used to hunt with the Elders who are deceased now*, reported Paul Jararuse, a participant from Kangiqsualujjuaq, who regularly visits Quaqtaq. *Last summer, July 2013, it was one month earlier. The migration of all marine mammals has changed now.”* Sammy Unatweenuk, a participant from Kangiqsualujjuaq, who visited Quaqtaq for beluga hunting in 2013 also explained that in the past, Atlantic walruses used to arrive a month after belugas, but nowadays they arrive a week later than belugas. “*It used to be belugas first, then walruses but nowadays all the animals come at the same time*,” explained Bobby Baron from Kangiqsualujjuaq.

## Discussion

In this study, Inuit Traditional and Local Ecological Knowledge (Inuit TEK/LEK), gathered through interviews with 33 local harvesters living in close relationship with walruses helped us understand changes in Atlantic walrus distribution and migration around Nunavik between the 1940s and the 2010s and their associations with changes in Arctic sea-ice patterns.

### Objective 1: Changes in Inuit land use patterns and harvesting practices

#### Fewer Atlantic walruses harvested

According to participants, fewer Atlantic walruses are being harvested nowadays by Nunavik residents. Relatively few communities from Nunavik are currently hunting Atlantic walruses, and generally only 30–60 walruses are hunted per year for the entire region of Nunavik, which corresponds to around half of the total number of walruses that used to be harvested in the 1980s (DFO 2002; Larrat et al. 2012). Participants of our study, as well as previous work (Dorais 1997; DFO 2013), suggest this decline results from a variety of factors including a decrease in the number of sled dogs to feed, but also the high costs of fuel. While walrus meat used to be an important part of the diet of Inuit sled dogs in Nunavik, the increased popularity of snowmobiles at the end of the 1960s has resulted in a decrease in the number of dogs to feed, and a concomitant decrease in the need for walrus meat (Dorais 1997; DFO 2013). Furthermore, according to reports made by Inuit from Nunavik, a series of dog slaughters were undertaken or ordered to be undertaken by federal and provincial government officials from the mid-1950s until the late 1960s (Laugrand and Oosten 2002; Qikiqtani Inuit Association 2014), although this has been denied by the Royal Canadian Mounted Police (http://www.makivik.org/dog-slaughter/). The high costs of hunting were also suggested to influence Pacific walruses hunting success in Alaskan communities (Huntington et al. 2013).

#### Smaller hunting areas and shorter hunting periods

We found that walrus hunters are now covering smaller hunting areas for shorter periods of time, which reduces the spatial and temporal range of their observations of Atlantic walruses around Nunavik. Such changes were partly explained by the settlement of Inuit that occurred mostly in the 1960s, which reduced the use of certain Inuit campsites, and thus the use of certain hunting areas (Dorais 1997). For example, in the 1940s–1950s, Inuit from the area of Quaqtaq were still nomadic, moving from their winter camp (now Quaqtaq) to their summer camps mostly located along the north-western coast of Ungava Bay (Dorais 1997). These former summer camps correspond to hunting areas that are nowadays rarely visited. Participants reported that the arrival of outboard motor canoes at the end of the 1960s (Dorais 1997; DFO 2013) can also partly explain changes in hunting patterns, and satellite phones more recently. These two technologies have decreased significantly the time needed to reach walrus hunting areas and also the time spent looking for walruses. Thus, hunters have become more efficient at harvesting walruses (DFO 2013).

Of the areas no longer used for walrus hunting, the area around Sleeper Islands ceased to be used for a different reason than the others. In the 1990s, hunters decided to stop hunting walruses there due to the high frequency of harvested animals testing positive for *Trichinella nativa*, a parasitic round worm that can infect humans and cause trichinellosis (Larrat et al. 2012). In 1997, the Nunavik Trichinellosis Prevention Program was created to prevent trichinellosis outbreaks associated with the consumption of raw or fermented walrus meat (Proulx et al. 2002; Larrat et al. 2012). This program still operates today and provides a 24-h diagnostic service and acts as a food safety-screening program for detection of *Trichinella nativa* larva in harvested walruses (Larrat et al. 2012). The last hunting trips made by Inukjuak walrus hunters to Sleeper Islands occurred in 2001 and 2003, and Inukjuak hunters began to travel to Salisbury and Nottingham Islands to go walrus hunting in 2005 (Manon Simard, personal communication). As a result, LEK of Atlantic walruses among local walrus harvesters around Sleeper Islands is no longer actively being generated and shared.

### Objective 2: Understand if the distribution and migration patterns of Atlantic walruses have changed around Nunavik

#### Changes in the distribution of Atlantic walruses around Nunavik

Our study highlighted changes in the distribution of Atlantic walruses around Nunavik, including abandoned haul-out sites. It is possible that, in the past, hunters harvested walruses directly on the land, disturbing them while they were resting, a strategy that has been criticized by hunters interviewed for this study. Indeed, previous studies revealed that walruses are sensitive to human disturbance, particularly, when it is affecting their land-based haul-out sites (Salter 1979; Metcalf and Robards 2008; DFO 2013).

It is also possible that walruses, which are bottom-feeding marine mammals, have modified the macrobenthic assemblage of the sea floor within a few kilometer radius around the islands (Oliver et al. 1985; Ray et al. 2006) making it difficult for them to harvest their major bivalve prey. This potential cause for a shift in walrus distribution away from haul-out sites corroborates an explanation given by a participant, who highlighted that species move from one place to another to look for food.

Though, it seems that abandoned haul-out sites can be re-colonized. Indeed, we found that Atlantic walruses had seasonally re-colonized areas such as some islands located near Ivujivik (e.g., Digges Islands Archipelago). Similarly, other walrus haul-out sites across the Canadian Arctic (e.g., near Chesterfield Inlet in Nunavut) that had been abandoned as a consequence of intensive killing are now being re-occupied by Atlantic walruses (DFO 2013).

### Atlantic walruses migrate around Nunavik one month earlier

In the 1970s, Dr. Louis-Jacques Dorais learned from Inuit that the first Atlantic walruses were arriving in the area of Quaqtaq at the end of July and beginning of August (Dorais 1997). Comparably, our study revealed that prior to the 1990s walruses used to arrive in the area of Quaqtaq in August. Nowadays, the first walruses are arriving at the end of June, thus a month earlier. Similarly, hunters from Alaska (USA) indicate that the spring migration of Pacific walruses occurs about a month earlier today than in past decades (MacCracken 2012).

As previously suggested (DFO 2013), participants explicated that Atlantic walruses are migrating earlier as the sea-ice is melting earlier. Changes in sea-ice cover reported by Inuit were consistent with Canadian ice service data, which showed a general decline in ice coverage within the Hudson Strait, which includes the Ungava Bay, on the first week of July between the 1970s and the 2010s (http://iceweb1.cis.ec.gc.ca/IceGraph/). Comparably to our study, several Arctic and sub-Arctic marine migrating species have advanced or delayed their migration as sea-ice retreats earlier in spring and forms later in fall (Ramp et al. 2015; Hauser et al. 2016; van Weelden et al. 2021). For example, as regional sea-ice freeze-up timing became later in the Beaufort, Chukchi, and Bering Seas, Pacific Chukchi beluga whales delayed their autumn migration by two to more than 4 weeks since the 1990s (Hauser et al. 2016).

Participants also reported that while in the past migrating species used to follow a predictable order of arrival, this is no longer the case. Dorais (1997) also reported that in the 1970s belugas were the first observed marine mammals by local Quaqtaq harvesters, followed one month later by Atlantic walruses. Around Quaqtaq, participants reported that Atlantic walruses arrive at the end of June and leave around mid-July. Interestingly, another LEK study reported, for similar decades, that belugas arrive around Quaqtaq at the end of April and leave around mid-July (Breton-Honeyman et al. 2016). It is possible that this indicated a 2-week overlap in the area of Quaqtaq for these two species, although quantitative data including dates of arrivals and departures for each year through several years would be needed to conclude. As belugas and Atlantic walruses prey on different species (Dehn et al. 2007; Breton-Honeyman et al. 2016), such potential temporal overlap should not cause food competition among them. On the contrary, scientific research has shown that while the timing of the northward migration of the fin whale (*Balaenoptera physalus*) and the humpback whale (*Megaptera novaeangliae*) to the Gulf of St. Lawrence has advanced in the season, both species maintained their approximate 2-week difference in arrival time and thus kept their temporal niche separation (Ramp et al. 2015).

## Conclusion

By analyzing qualitative data from 33 interviews with local walrus hunters and Elders as part of a larger project on Atlantic walruses in Nunavik (Martinez-Levasseur et al. 2016, 2017, 2020), we found that the location and size of walrus hunting areas, as well as the time spent by hunters to look for walruses in those areas has changed over participants’ lifetime. This is likely due to a combination of factors, including Inuit settlement in the 1960s and the arrival, over the last decades, of technologies facilitating walrus harvesting such as outboard motor canoe or satellite phones. Our study not only highlighted important changes in walrus hunting practices and locations among Nunavik Inuit communities since the 1940s but also sheds light on the importance of documenting temporal and spatial changes in Inuit land use patterns and harvesting practices when studying Arctic species described by Inuit TEK/LEK. Understanding spatial and temporal changes in Inuit land use patterns and harvesting practices was essential to interpreting spatial and temporal changes in Atlantic walrus distribution using Inuit TEK/LEK, which was the main goal of our study. We found that fewer walruses are currently being harvested around Nunavik when compared with pre-1990 levels, and that some walruses are now re-occupying areas they had previously abandoned. Furthermore, the timing of the spring migration of Atlantic walruses traveling through the area of Quaqtaq has changed, likely due to changes in Arctic sea-ice patterns. Indeed, we learnt from Inuit observations that Atlantic walruses that follow the melting ice to travel are now migrating through the area of Quaqtaq one month earlier than prior to the 1990s. Those important changes in Atlantic walrus distribution and migration were only possible to understand using rigorous methods to interpret Inuit TEK/LEK.

## Supplementary Information

Below is the link to the electronic supplementary material.

Supplementary file1 (PDF 354 KB)

Supplementary file2 (PDF 405 KB)
