# Supplementary material for: New migration and distribution patterns of Atlantic walruses (Odobenus rosmarus rosmarus) around Nunavik (Québec, Canada) identified using Inuit Knowledge
Source: Polar Biol. 2021 Aug 6;44(9):1833–45. doi: 10.1007/s00300-021-02920-6 (PMC8550009; doi:10.1007/s00300-021-02920-6)
Supplement: Supplementary file 1 — Supplementary file1 (PDF 354 KB) [file 300_2021_2920_MOESM1_ESM.pdf]

ELECTRONIC SUPPLEMENTARY MATERIAL - 1

New migration and distribution patterns of Atlantic walruses (*Odobenus rosmarus rosmarus*) around Nunavik (Québec, Canada) identified using Inuit Knowledge

Laura M. Martinez-Levasseur[1,2,3,*], Chris M. Furgal[2], Mike O. Hammill[4], Dominique A. Henri[3] and Gary Burness[1]

[1] Department of Biology, Trent University, Peterborough, Ontario, K9L 0G2, Canada
[2] Indigenous Environmental Studies & Sciences Program, Trent University, Peterborough, Ontario, K9L 0G2, Canada
[3] Wildlife Research Division, Environment and Climate Change Canada, Montréal, Québec, H2Y 2E7, Canada
[4] Maurice Lamontagne Institute, Fisheries and Oceans Canada, Mont-Joli, Québec, G5H 3Z4, Canada

*Corresponding author
E-mail: lmmartinezlevasseur@gmail.com

SUPPLEMENTARY FIGURES

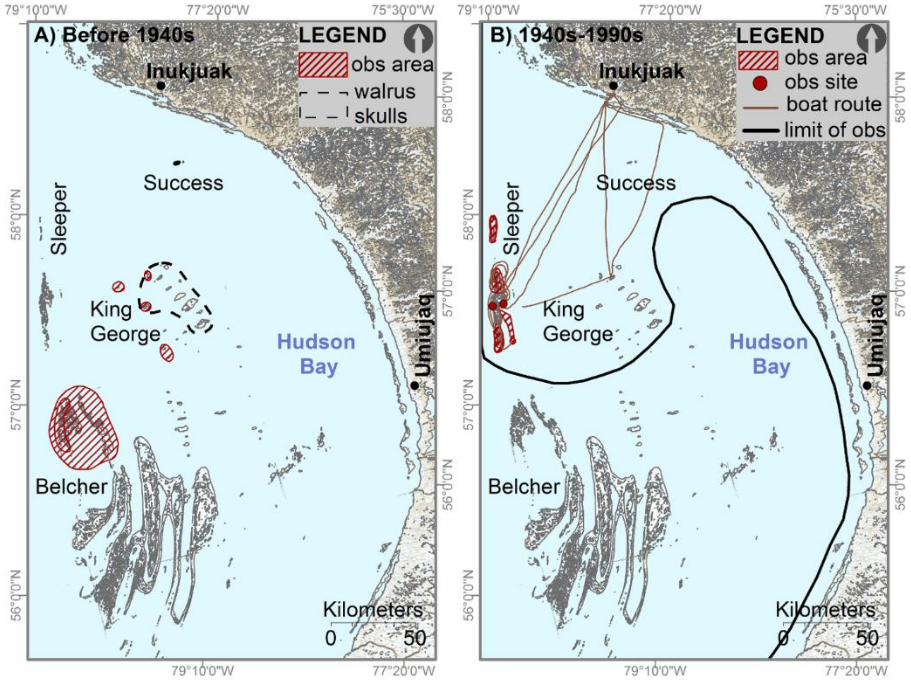

Figure S1. Distribution of Atlantic walruses (*Odobenus rosmarus rosmarus*) during fall (Sept-Oct) in western Hudson Bay based on Inuit knowledge and observations. (A) Indirect walrus observations areas (hatched areas) prior to the 1940s, as reported and drawn by Inukjuak participants (corresponding to the observations transmitted to them from their ancestors). (B) Direct walrus observations areas for the 1940s-1990s period, as reported and drawn by Inukjuak participants. Each hatched area and point was drawn by one participant and corresponds to areas or sites where walruses had been observed or harvested by this participant. Each thin line was drawn by a single participant and represents one of the routes (one expedition per year) taken by the community boat to go walrus hunting between the 1940s and the 1990s. The same line can represent the path taken by the community boat several times. The thick lines correspond to the limits of the common area of observation of all participants. This figure combines digitalized data collected from seven Inukjuak participants in 2013-2014.

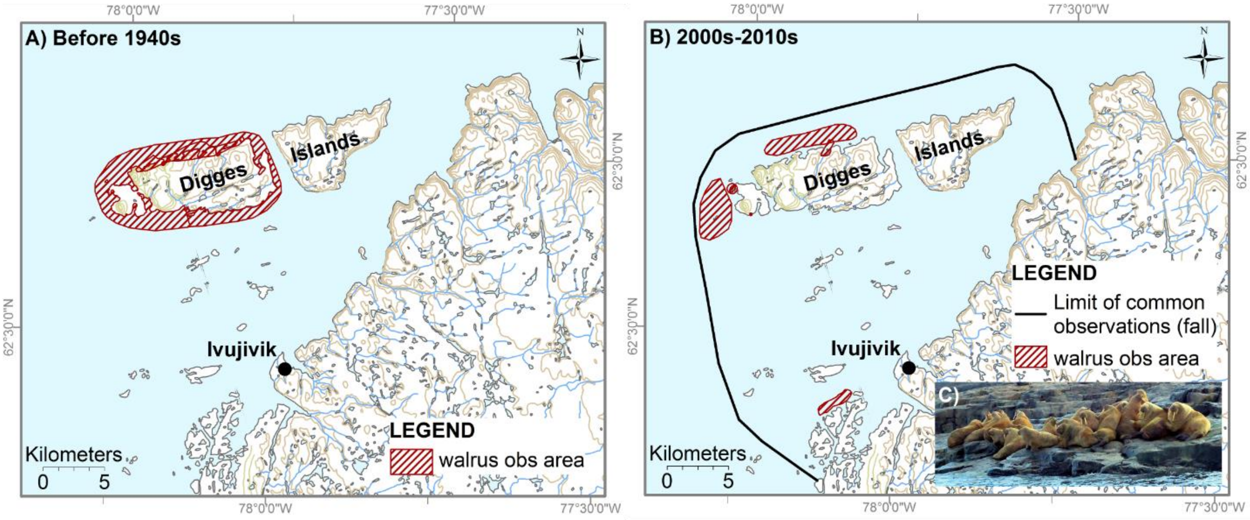

Figure S2. Distribution of Atlantic walruses (*Odobenus rosmarus rosmarus*) during fall (September to mid-December) around Ivujivik based on Inuit knowledge and observations. (A) Indirect walrus observations areas (hatched areas) prior to the 1940s, as reported and drawn by two Ivujivik participants (corresponding to the observations transmitted to them from their ancestors). No common area of observation could be documented for this period. (B) Direct walrus observations areas for the 2000s-2010s period in the fall, as reported and drawn by four Ivujivik participants. Each hatched area has been drawn by one participant and correspond to areas where walruses has been observed by this participant. The black lines correspond to the limits of the common areas of observations of all participants. (C) Evidence of basking walruses observed in October 2013 on the north-western shores of the Digges Islands Archipelago (photo: Charlie Paningayak). This figure combines the digitalized data collected from Ivujivik participants in 2013-2014.
