# Supplementary material for: New migration and distribution patterns of Atlantic walruses (Odobenus rosmarus rosmarus) around Nunavik (Québec, Canada) identified using Inuit Knowledge
Source: Polar Biol. 2021 Aug 6;44(9):1833–45. doi: 10.1007/s00300-021-02920-6 (PMC8550009; doi:10.1007/s00300-021-02920-6)
Supplement: Supplementary file 2 — Supplementary file2 (PDF 405 KB) [file 300_2021_2920_MOESM2_ESM.pdf]

ELECTRONIC SUPPLEMENTARY MATERIAL - 2

New migration and distribution patterns of Atlantic walruses (*Odobenus rosmarus rosmarus*) around Nunavik (Québec, Canada) identified using Inuit Knowledge

Laura M. Martinez-Levasseur[1,2,3,*], Chris M. Furgal[2], Mike O. Hammill[4], Dominique A. Henri[3] and Gary Burness[1]

[1] Department of Biology, Trent University, Peterborough, Ontario, K9L 0G2, Canada
[2] Indigenous Environmental Studies & Sciences Program, Trent University, Peterborough, Ontario, K9L 0G2, Canada
[3] Wildlife Research Division, Environment and Climate Change Canada, Montréal, Québec, H2Y 2E7, Canada
[4] Maurice Lamontagne Institute, Fisheries and Oceans Canada, Mont-Joli, Québec, G5H 3Z4, Canada

*Corresponding author
E-mail: lmmartinezlevasseur@gmail.com

RESULTS ON ATLANTIC WALRUS (*ODOBENUS ROSMARUS ROSMARUS*) MIGRATION AROUND NUNAVIK (HUDSON STRAIT STOCK)

First, we acknowledge that TEK/LEK on Atlantic walrus (*Odobenus rosmarus rosmarus*) migration represent the path taken by only a portion of the corresponding walrus stock, specifically those travelling within participants' common areas of observations (Martinez-Levasseur et al. 2017).

The migrating Atlantic walruses observed and reported by Kangiqsualujjuaq participants (Killiniq Island, 1940s-1960s) and Quaqtaq participants (Quaqtaq area, 1940s-2010s) are those from the Hudson Strait stock (see map in Stewart 2008). According to participants, the general path taken during spring migration by a portion of the Atlantic walruses from

this stock begins along the Labrador coast at the end of April, surrounds the Labrador coast in May-June, crosses the Ungava Bay to reach the area of Quaqtaq at the end of June and ends on the Islands of Nottingham and Salisbury (Fig. S1), where Ivujivik participants harvest walruses in the fall (September-October). Combining information from these three communities allowed us to cover a large part of the migration of Atlantic walruses from the Hudson Strait stock (Stewart 2008).

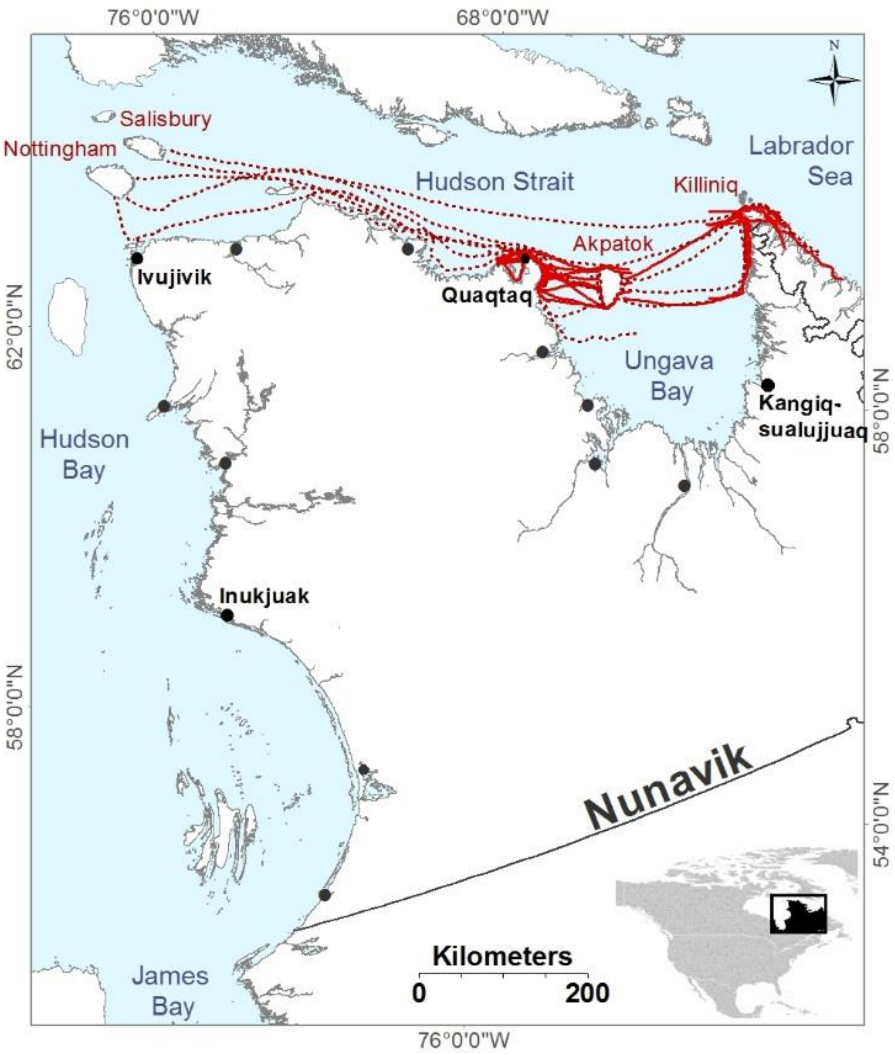

Figure S1. Atlantic walrus (*Odobenus rosmarus rosmarus*) spring migration routes along the eastern and northern coasts of Nunavik, as drawn by Quaqtaq participants (1940s-2010s) and Kangiqsualujjuaq participants (1940s-1960s). The full lines represent the knowledge held by participants and drawn on local maps (scales between 1:100,000 and 1:280,000). The dotted lines reported the knowledge drawn on the regional map (scale: 1:2,000,000), which only represents an

approximation of the general path taken by Atlantic walruses during their spring migration, as highlighted by participants. This figure was created using the geographic information system software ArcMap 10.4.1. Digital vector datasets: RNCan-National Topographic Database.

From the Labrador coast to Akpatok Island

After spending winter along the Labrador coast, Atlantic walruses start their spring migration at the end of April, reported one participant. Walruses follow the Labrador coast northward and used to be observed near Killiniq Island on the side of the Labrador Sea between May and mid-June, explained 75% of participants (6 out of 8). Then, walruses are mainly observed along the East coast of Ungava Bay and then on Akpatok Island (Fig. S1). Between these two areas, walruses were rarely observed, informed participants.

In total, 94% of participants (15 out of 16) explained that the timing of walrus spring migration is associated with the quantity and condition of ice. Walruses travel after most ice packs had moved, generally pushed offshore by the wind, explained participants. Seventy five percent of participants (12 out of 16) reported that walruses use the tide and the costal counter current, when migrating. *"The walruses we saw* [McLelan Strait, situated south of Killiniq Island] *were travelling against the* [main] *current. That's why maybe they swim very close to the land to avoid the current.* […] *There is heavy current on McLelan but close to the coast it is almost a reverse effect with the tide"*, explained Sammy Unatweenuk from Kangiqsualujjuaq. The distance to the coast is an important variable for walrus migration, as reported by 81% of participants (13 out of 16). However, walruses can travel offshore when there is too much ice along the coast, reported Kangiqsualujjuaq participants. Bobby Baron from Kangiqsualujjuaq explained that when travelling from Killiniq towards Quaqtaq, walruses cross Ungava Bay instead of following the coast. Kangiqsualujjuaq participants also reported that walruses cross directly to Quaqtaq without stopping on Akpatok if there is too much ice in Ungava Bay. Finally, 13% of participants (2 out of 16) added that walruses can modify their route if they encounter predators or if there is too much human disturbance.

Sixty seven percent of participants from Quaqtaq and Kangiqsualujjuaq (8 out of 12) explained that walruses travel in groups of less than five individuals or in groups of 5-15 individuals. Two participants reported observing larger groups, including groups of more than 50 walruses, although they added that this was rare. The first groups of migrating walruses are females with their offspring, reported 67% of Quaqtaq participants (4 out of 6).

From Akpatok Island to Diana Bay

Walruses take any route between Akpatok and the western coast of Ungava Bay (Fig. S2), explained Quaqtaq participants. Walruses travel near Quaqtaq during two to four weeks between the end of June and mid- July. Participants clarified that although the first walruses are observed in June, most walruses are seen during the first two weeks of July. Walruses are then reported travelling northward along the coast up to Quaqtaq, where they cross Diana Bay (*Tuvaaluk*) without going through the bay (Fig. S2). During the validation workshop, participants explained that the routes taken by walruses in the North of Diana Bay were unsure as it is an area rarely explored by hunters (not included in hunters' common areas of observations).

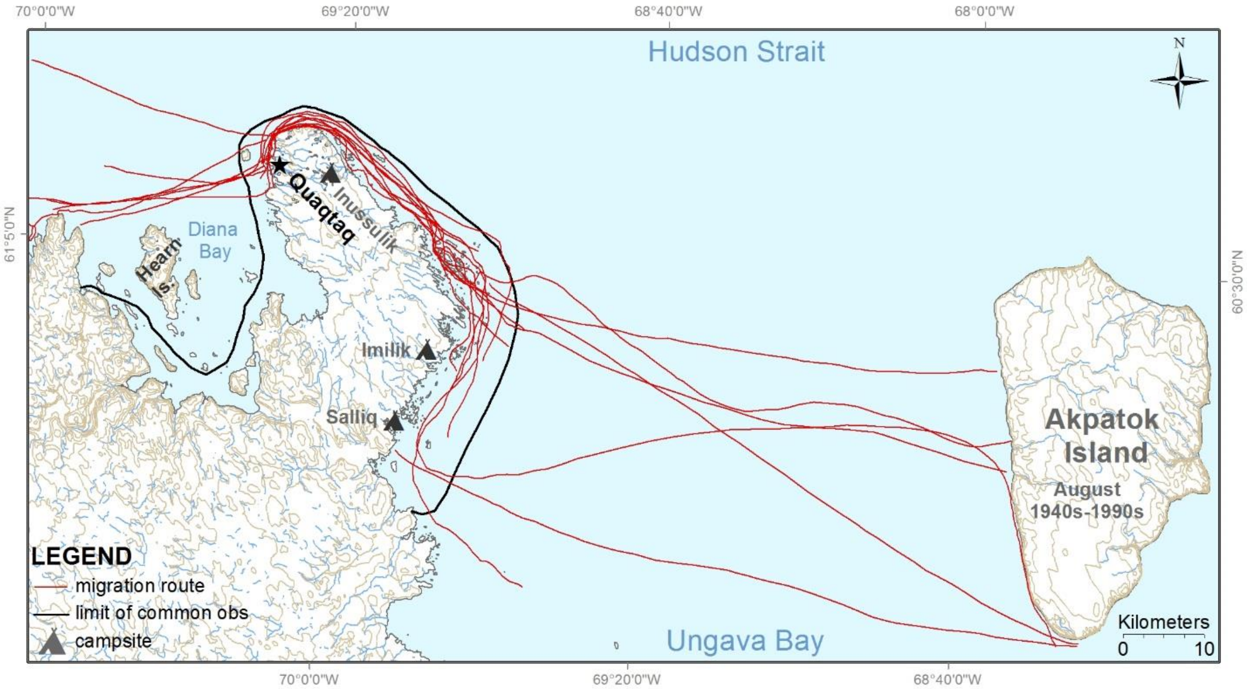

Figure S2. Spring migration paths taken by Atlantic walrus (*Odobenus rosmarus rosmarus*) from Akpatok to Quaqtaq (participants observations dated between 1940s and 1990s). The black lines correspond to the limits of the common areas of observations of participants since 1990s. This figure was created using the geographic information system software ArcMap 10.4.1. Digital vector datasets: RNCan-National Topographic Database.

From Diana Bay to Nottingham and Salisbury Islands

As the area from Diana Bay to Nottingham and Salisbury Islands was outside of participants' common areas of observations (Martinez-Levasseur et al. 2017), we could not document the path taken by walruses between these two areas.

Nottingham and Salisbury Islands (walrus resting summer ground)

Ivujivik hunters have been harvesting Atlantic walruses near their resting grounds around Nottingham and Salisbury Islands in the fall (September-October). They reported that the groups of walruses observed are formed by males and females. Offspring are observed anywhere around the islands, particularly on basking areas, as reported by 75% of Ivujivik participants (6 out of 8). Walrus groups are generally made of around 30 individuals, as

stated by two Ivujivik Elders, who added that they had never observed large groups of several hundred of walruses. Two active hunters, including the current captain of the community boat, reported that they once saw more than a hundred walruses on a small island called *Akulliq*, near Salisbury Island.

Participants explained that there is always one walrus on the top of the island. If danger is perceived such as a coming boat, this walrus makes noises and all the walruses flee. *"Walruses have a scout who is usually on top of the island, searching for any danger, and if it sees any danger it is able to call out to the population"*, explained Quitsaq Tarriasuk from Ivujivik. Adamie Kalingo from Ivujivik added: *"The females are protecting their young close to the coast. Whereas the males and the scout are up higher. If they need to escape quickly the young and the females are ready to flee the basking area."*

Among Ivujivik participants, 86% (6 out of 7) reported that walrus basking areas are generally surrounded by deep water corresponding to waters of about 50-70 feet deep (15-20 meters). Participants explained that walruses prefer islands surrounded by deep water for feeding. Furthermore, walruses have to dive deep to avoid attacks of polar bears, which can only dive to a maximum of 10 feet (i.e. around three meters). Walruses were rarely observed in areas too exposed to the wavy sea and areas with lots of current, such as the north of Nottingham, as explained by 43% of Ivujivik participants (3 out of 7). *"When it is warmer climate during the summer, walruses will abandon the island for a while, the Island of Tujjaat* [Nottingham]", explained Quitsaq Tarriasuk from Ivujivik, who was agreed by 43% of Ivujivik participants (3 out of 7). Finally, 43% of Ivujivik participants reported that walrus avoid cliffy coasts.

Return migration

Participants from Kangiqsualujjuaq and Quaqtaq explained that Atlantic walruses use the same migration routes in the fall to return to overwintering grounds along the coast of Labrador. However, observations in the fall were rare, mostly due to the bad weather conditions that do not allow hunters to go boating. *"On their return we don't see them. We*

don't have access to the sea in the fall, it is impossible by boat and too dangerous by ski-doos, explained Sammy Unatweenuk from Kangisualujjuaq. *It is very rare to see walruses in the fall but they need to come back before the ice froze, so before December."* Walrus direct observations between October and the beginning of December mostly occurred around living areas corresponding to the participants' area of knowledge in the fall (Martinez-Levasseur et al. 2017). Within this area, Quaqtaq participants reported that walruses are travelling closer to the land on their return, sometimes following the coast of Diana Bay. *"Walruses are coming through here* [Quaqtaq Point]*, which is what we call utarkiavik, waiting place for the animals.* […] *We can use that point from now* [end June] *until October, November,* explained David Okpik from Quaqtaq. *If the fast ice is formed* [sea-ice fastened to the coastline], *we move to the island instead* [to Hearn Island to observe walruses coming back]*"*.

REFERENCES

Martinez-Levasseur LM, Furgal CM, Hammill MO, Burness G (2017) Challenges and strategies when mapping local ecological knowledge in the Canadian Arctic: the importance of defining the geographic limits of participants' common areas of observations. Polar Biol 0:1–13. https://doi.org/10.1007/s00300-016-2071-2

Stewart REA (2008) Redefining walrus stocks in Canada. Arctic 61:292–308
